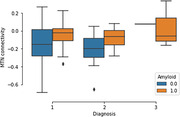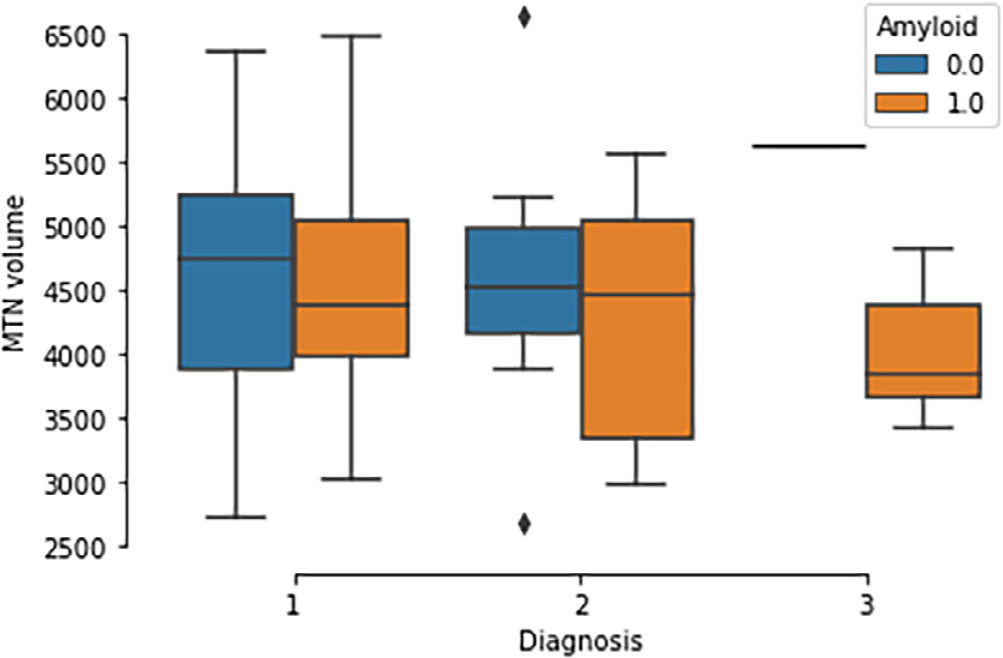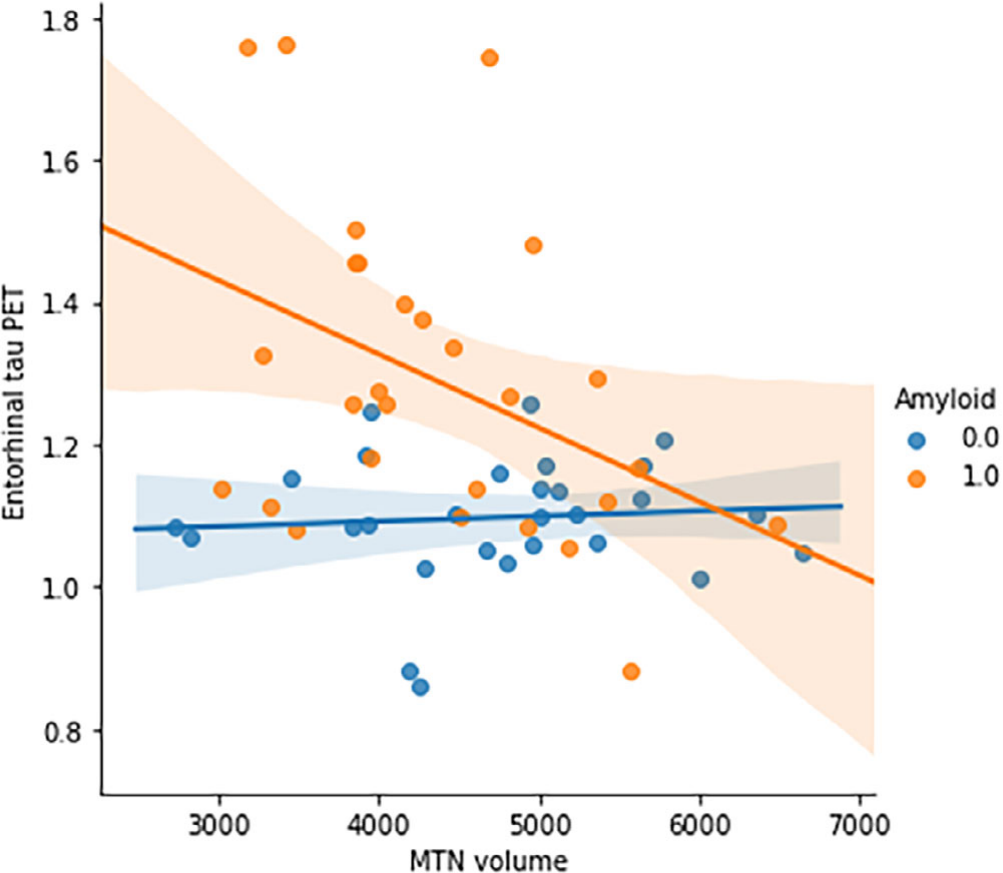# Changes in individualized functional brain networks at the preclinical stage of Alzheimer’s disease

**DOI:** 10.1002/alz.087789

**Published:** 2025-01-09

**Authors:** Ioannis Pappas, Cally Xiao, Arthur W. Toga

**Affiliations:** ^1^ Laboratory of Neuro Imaging, Stevens Neuroimaging and Informatics Institute, Keck School of Medicine, University of Southern California, Los Angeles, CA USA

## Abstract

**Background:**

Prior work in preclinical Alzheimer’s disease has shown different phases of hyper and hypo connectivity especially within the default mode network (DMN) (Schultz et al., 2017). However, these analyses often rely on group templates that estimate network connectivity based on predefined parcels, potentially obscuring individual variability. New methods allow us to estimate networks at the individual level and these networks show individual‐specific variation in terms of their connectivity, size, and spatial arrangement (Kong et al., 2019; Gordon et al., 2017).

**Method:**

Individualized networks were extracted data from 121 subjects from the Alzheimer’s Disease Neuroimaging Initiative (ADNI) dataset based on availability of functional MRI, amyloid, and tau PET data. Functional MRI data was processed following methods by (Yeo et al., 2011) pipeline. We used methods from (Kong et al., 2019) to obtain individual‐specific networks using the 17‐network parcellation as a prior. In this work we focused on the medial temporal network subcomponent of the DMN (MTN) and examined its connectivity and volume. Amyloid positivity and tau PET standard uptake value ratios (SUVRs) (entorhinal) were derived from the ADNI data.

**Result:**

Out of the initial cohort, 72 individuals had usable fMRI data. MTN connectivity was significantly higher in amyloid positive (A+) versus amyloid negative (A‐) individuals with no cognitive impairment (p<0.01). MTN network volume did not change between A+ and A‐ individuals. When looking at the relationship between network markers and tau PET signal, we found that MTN volume was negatively correlated with tau PET SUVRS in entorhinal cortex (r = ‐0.3; p=0.01). MTN connectivity was not related to entorhinal tau PET signal. No interaction effects were observed between amyloid positivity and tau PET signal when predicting connectivity or volume. Exploratory analysis revealed that baseline MTN connectivity was positively correlated with change in entorhinal tau PET signal over two years.

**Conclusion:**

These results suggest that individualized networks are sensitive to amyloid and tau biomarkers in preclinical Alzheimer’s disease. Network connectivity seems to track amyloid burden while network volume is more related to tau PET signal. Overall, individualized approaches can reveal subtle network changes that can be related to disease biomarkers.